# Design, Fabrication and Failure Analysis of Stretchable Electrical Routings

**DOI:** 10.3390/s140711855

**Published:** 2014-07-04

**Authors:** Hong Hocheng, Chao-Ming Chen

**Affiliations:** Department of Power Mechanical Engineering, National Tsinghua University, No.101, Section 2 Kuang Fu Road, Hsinchu 30013, Taiwan; E-Mail: d947729@oz.nthu.edu.tw

**Keywords:** microelectromechanical systems (MEMS), stretchable, routing, strain, failure

## Abstract

Stretchable microelectromechanical systems (MEMS) possess higher mechanical deformability and adaptability than devices based on conventional solid and flexible substrates, hence they are particularly desirable for biomedical, optoelectronic, textile and other innovative applications. The stretchability performance can be evaluated by the failure strain of the embedded routing and the strain applied to the elastomeric substrate. The routings are divided into five forms according to their geometry: straight; wavy; wrinkly; island-bridge; and conductive-elastomeric. These designs are reviewed and their resistance-to-failure performance is investigated. The failure modeling, numerical analysis, and fabrication of routings are presented. The current review concludes with the essential factors of the stretchable electrical routing for achieving high performance, including routing angle, width and thickness. The future challenges of device integration and reliability assessment of the stretchable routings are addressed.

## 1. Introduction

Stretchable electrical devices, unlike flexible electrical devices, possess high mechanical stretchability, and are more appropriately applied in special fields, such as biomedical and textile applications. The technology of making stretchable electrical routings is most desired in body health monitoring devices. It also helps the development of prosthetic limbs providing human-like sensation simulations. In recent research, specific designs and applications have been studied and keep advancing.

**Figure 1.**
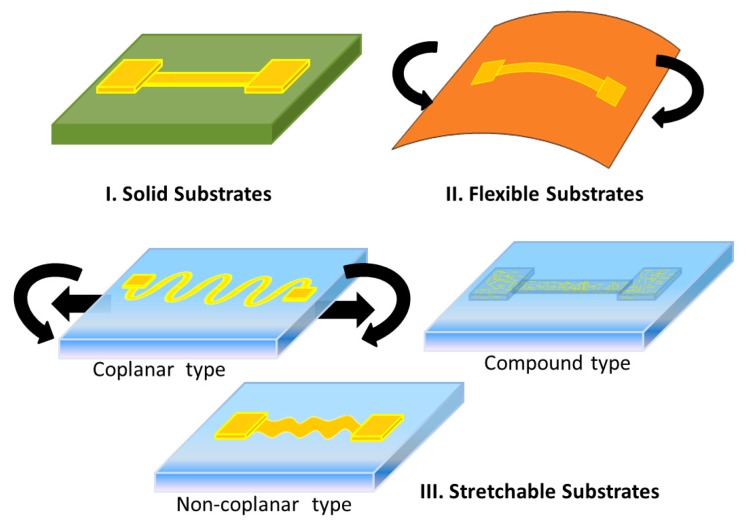
Solid substrates, flexible substrates, and stretchable substrates.

The concepts of typical common substrates are shown in Figure 1. Traditional electronic devices use solid substrates due to their high strength against bending and impacts, and they possesses the highest reliability among the three. Flexible substrates show limited bendability and little stretch-ability when the environment requires it, although the stretchable property is not normally considered as part of their performance features. Hence, if one folds flexible circuits for a thousand cycles, their lifetime will decrease drastically. Stretchable circuits, divided generally into three types, can sustain large bending and stretching actions, and their applications are different from the other two because they can be placed on any non-planar surface. However, their lifetime is the lowest among the three, because of the working conditions—being bent and stretched constantly.

**Figure 2.**
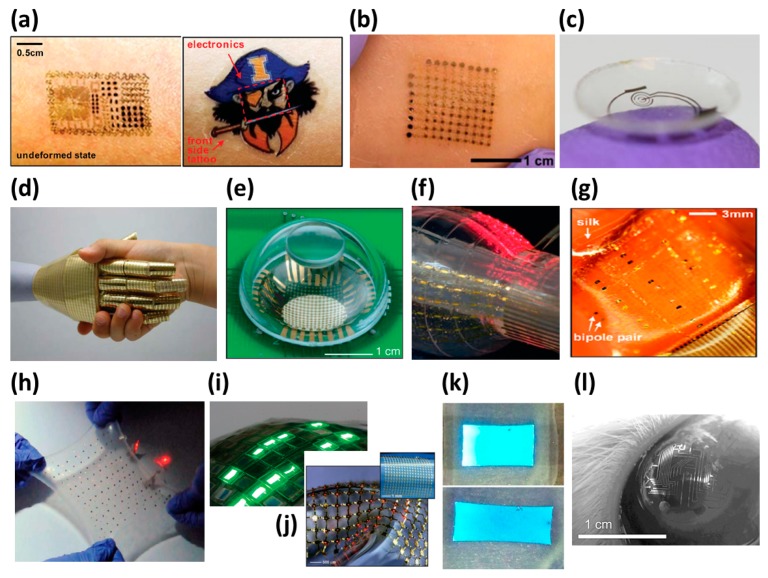
Application concepts of stretchable devices. (**a**) Multifunctional epidermal electronics integrated with a transferred tattoo on skin. Reproduced with permission from [1]; published by AAAS, 2011. (**b**) Electronic sheets determining the hydration of body. Reproduced with permission from [2]; published by IEEE, 2013. (**c**) Smart contact lens with a function of measuring the glucose level. Reproduced with permission from [3]; published by Elsevier, 2011. (**d**) Electronic artificial skin. Reproduced with permission from [4]; published by John Wiley and Sons, 2010. (**e**) Electronic eyes. Reproduced with permission from [6]; published by Nature Publishing Group, 2008. (**f**) Interventional catheter with tactile sensing. Reproduced with permission from [7]; published by Nature Publishing Group, 2011. (**g**) Epicardial electrogram mapping on a silk substrate. Reproduced with permission from [12]; published by the National Academy of Sciences, U.S.A. 2012. (**h**) Stretchable batteries. Reproduced with permission from [13]; published by Nature Publishing Group, 2013. (**i**) Stretchable organic light-emitting diode (OLED) arrays. Reproduced with permission from [14]; published by Nature Publishing Group, 2009. (**j**) Stretchable inorganic light-emitting diode (ILED) arrays. Reproduced with permission from [15]; published by AAAS, 2009. (**k**) Fully stretchable Stretchable organic light-emitting diode (OLED). Reproduced with permission from [16]; published by John Wiley and Sons, 2011. (**l**) Contact lenses with imprinted electronic circuits and lights. Reproduced with permission from [17]; published by IEEE, 2008.

Stretchable electronics can be applied in specific fields, as shown in Figure 2. For the human body, a health monitor capable of detecting temperature, activity, and electrocardiography (ECG) signals was developed as an epidermal device which could be hidden and protected by a temporary transfer tattoo [1] (Figure 2a). Similar technology is also applied in an electronic sheet [2] (Figure 2b) which determines the hydration of the body during the training of an athlete and gives periodical reminders to drink for maintaining proper hydration. Different from previous devices in style, a smart contact lens developed with an embedded sensor can measure the glucose levels of diabetics to help their daily health tracking [3] (Figure 2c). In the field of prosthetic limbs, electronic artificial skins [4,5] (Figure 2d) and electronic eyes [6] (Figure 2e) have been studied to help vision and touch to become possible. In an application directly into a human organ, the coronary angioplasty procedure works by an interventional catheter with a tiny balloon placed into a location to compress the fatty build-up against the artery wall. The balloon part of an interventional catheter integrating an array of touching sensors [7] (Figure 2f) can detect the proper contact situation and help the doctors gain more control while inflating the balloon. The curvilinear electronics technology [8] proves a stretchable circuit mesh placed on a non-planar surface, such as the skin of a heart. A stretchable microelectrode array (SMEA) [9–11] applied in electrocorticography (ECoG) is used to detect the activities of the human brain by monitoring the change of brain electrical impulses [12] (Figure 2g). The use of stretchable wires and microelectrodes integrated into a film helps increase the resolution of detection of the behavior of a fixed predetermined brain section. In the energy part of stretchable electronics, a battery array connected by serpentine wires has been produced recently [13] (Figure 2h). It supports the possibility of devices that can work inside the human body in the future. In the field of information display, stretchable displays can be made with organic light-emitting diode (OLED) or inorganic light-emitting diode (ILED) arrays in a film, as shown in Figure 2i [14] and Figure 2j [15], or fully stretchable OLEDs [16] (Figure 2k). A contact-glass type embedded μ-LED is also under study [17] (Figure 2l).

Flexible devices have become popular in modern medical and display development, and lots of research and prototypes have been presented, and a flexible smartphone (the G Flex developed by LG (Seoul, Korea)) is already sold on the market. The stretchable devices have more advantages in the specific applications mentioned above. The recent development of stretchable electrical routings just starts to explore all the microfabrications with better performance and reliability that are possible. The typical technologies are discussed in this paper—electrical routing designs, failure analysis and processes.

## 2. Design and Fabrication of Stretchable Routing

Stretchable routings need a specific physical electronic conductor structure prepared by processing technology and designed for stretchability. For pure solid metals with stretchable behavior, the wires will go with serpentine geometry. Whether the serpentine path on a substrate is coplanar or non-coplanar; both types can serve the purpose—provide stretchability. Two essential methods to make a stretchable routing on stretchable substrates are presented: routing design and the processes; the coplanar type is often made by routing design and the non-coplanar type is usually made by using particular processes.

### 2.1. Routing Design

**Figure 3.**
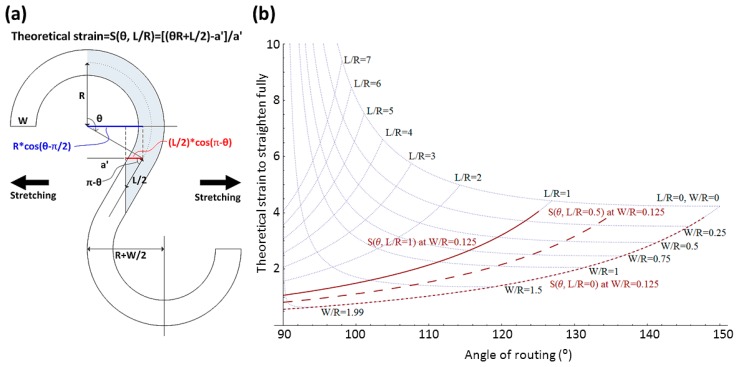
(**a**) Concepts of a serpentine routing; and (**b**) Design map of routing with factors of θ, *W*/*R*, and *L*/*R*.

A coplanar serpentine routing as shown in Figure 3a has the parameters of *R* (radius of arc), θ (arc angle), *L* (straight section of routing), and *W* (width of routing).

There is a geometric function described by Equation (1) if adjacent patterns are not overlapped. The boundary conditions also can be obtained by *L* = 0 and *L* ≠ 0:

(1) $$\begin{array}{c} R\cos(\theta -\frac{\pi }{2})-\frac{L}{2}\cos(\pi -\theta )\geq \frac{1}{2}(R+\frac{W}{2})\\ \text{Boundary condition}\;1(\text{if}\;L=0):\theta \leq \pi -\arcsin(\frac{1}{2}+\frac{1}{4}\frac{W}{R})\\ \text{Boundary condition}\;2(\text{if}\;L\neq 0):\frac{L}{R}\leq 2\sec\theta (\frac{1}{2}+\frac{1}{4}\frac{W}{R}-\sin\theta ) \end{array}$$

The theoretical strain of a coplanar serpentine routing stretched fully can be expressed by Equation (2):

(2) $$\varepsilon_{\text{coplanar type}}\left(\theta ,\frac{L}{R}\right)=\frac{2\theta +\frac{L}{R}}{2\sin\theta +\frac{L}{R}\cos\theta }-1$$

Since ε_coplanar type_ is a function of θ and *L*/*R*, the result with the boundary conditions is shown in Figure 3b. It can decide the theoretical strain of an initial routing design, *i.e.*, 57% strain at ε_coplanar type_ (90°, 0) [18], 142% strain at ε_coplanar type_ (120°, 0) [19], or 280% strain at ε_coplanar type_ (140°, 0). Further, 150° is the theoretical geometric limit if *L*/*R* = 0 and *W*/*R* = 0. Notice that Equation (2) doesn't include failure type detection and the natural stretchability based on the materials of elastic conductors. Equation (2) with the boundary conditions is a pre-design tool to know the initial limitations of a designed routing before any simulations and experiments. The strain of a non-coplanar wrinkly routing is expressed by Equation (3) [20]:

(3) $$\varepsilon_{\text{non}-\text{coplanar type}}=\frac{L_{\max}-L_{\text{o}}}{L_{\text{o}}},\text{where}\;L_{\max}=2N\times \int_{0}^{\pi }\sqrt{1+{\left(\frac{2\pi A}{\lambda }\cos\frac{2\pi x}{\lambda }\right)}^{2}}dx$$

Here *L*_max_ is the real length of a wrinkly wire, *L*_o_ is the length along the track direction, *N* is the number of waves along the length direction, λ is the wavelength, *A* is the amplitude of waves. The values of amplitude and wavelength of routing are highly related with the elastic modulus of wires and substrates and the pre-strained level of substrates as shown in Equations (4) and (5) [21]:

(4) $$\lambda =\frac{2\pi h_{\text{f}}}{(1+\varepsilon_{\text{pre}}){\left(1+\xi \right)}^{\frac{1}{3}}}{\left[\frac{E_{f}(1-\upsilon_{s}^{2})}{3E_{s}(1-\upsilon_{f}^{2})}\right]}^{\frac{1}{3}}$$

(5) $$A=\frac{h_{f}}{\sqrt{1+\varepsilon_{\text{pre}}}{\left(1+\xi \right)}^{\frac{1}{3}}}\sqrt{\frac{\varepsilon_{\text{pre}}}{\varepsilon_{\text{c}}}-1}$$

where *E* is the Young's modulus, *v* is the Poisson's ratio, *s* means the substrate, *f* means the stiff film on the substrate, *h*_f_ is the thickness of stiff film, ε_pre_ is the prestrain of the substrate, $\xi =\frac{5}{32}\varepsilon_{\text{pre}}(1+\varepsilon_{\text{pre}})$ expresses the large deformation and geometrical nonlinearity in the substrate, and $\varepsilon_{c}=\frac{1}{4}{\left[\frac{3E_{\text{s}}(1-\upsilon_{\text{f}}^{2})}{E_{\text{f}}(1-\upsilon_{\text{s}}^{2})}\right]}^{\frac{2}{3}}$ is the critical buckling strain.

**Figure 4.**
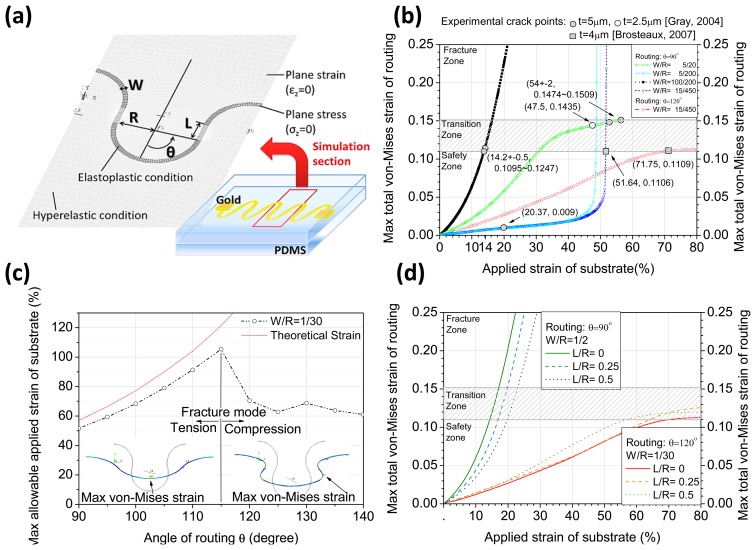
Simulation of two-dimensional stretchable routing in a coplanar plane: (**a**) Finite element model; (**b**) Simulation of stretch test compared with experimental results [18,19]; (**c**) Effects of varying angle of routing in stretch test; and (**d**) Effects of varying *L*/*R* ratio of routing in stretch test. Reproduced with permission from [22]; published by JSAP, 2013.

After the routing geometry design, building the simulation model and checking the real strain of a routing could find us a possible way to identify what parameters should be studied for safe use. The simulation model [22] of a stretchable routing on a coplanar plane shown in Figure 4a is constructed by mechanical assumptions from the interconnection section of a common elastomeric electronic device. It reveals the influence of width-to-radius of curvature (*W*/*R*) ratio, connect-to-radius of curvature (*L*/*R*) ratio, and angle of routing in pure tension mode. The material properties of the substrate (PDMS) are based on the Mooney-Rivlin hyperelastic model (mixed ratio 5:1) [23], and the nonlinear behavior of the routing wire (gold) is considered in use of the true stress-strain characteristics derived from experimental data [24]. Figure 4b shows the strain growth of the routing while an external strain is applied. One finds that the maximum applied strain [18,19] pulls the wavy routings to a strain of 11%–15%. It indicates that routing fracture will occur because the maximum allowable strain is 10.6% in tension tests [24]. The simulated situation is close to the real phenomenon; hence the interval of the transition zone is proposed to be defined at 11%–15% based on the above-mentioned results. Figure 4c shows that the stretchability performance, with a fixed *W*/*R* ratio, increases linearly before θ = 115°, reduces drastically at θ = 120°, and remains low in the rest of the region. This is attributed to the fact that the pulling effect from the substrate is magnified with larger routing angles, and the fracture mode is changed from tension to compression. The effect of *L*/*R* ratio of routing of two cases is shown in Figure 4d, and one finds that the higher the *L*/*R* ratio of routing is, the more evident a strain shift is shown due to the difference of their fracture mode. The results indicate that the stretchability increases by reducing the *W*/*R* ratio, but that it not always increases by increasing the angle of routing due to the pulling effect from the substrate. *L*/*R* ratio of routing will enhance either the advantages or disadvantages of designated routings, and hence should be designed carefully.

**Figure 5.**
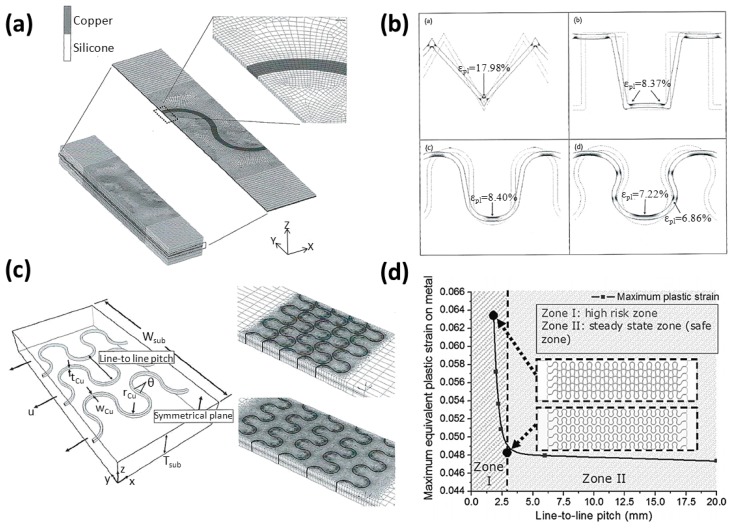
Simulation of three-dimensional stretchable routings: (**a**) Simulation model of a single routing; (**b**) Effects of varying patterns of routing in stretch test; (**c**) Simulation model of parallel routings; and (**d**) Effects of varying pitch between parallel routings in stretch test. (**a**,**b**) are reproduced with permission from [25]; published by John Wiley and Sons, 2012. (**c**,**d**) are reproduced with permission from [26]; published by IOP Publishing, 2008.

In three-dimensional simulation models as shown in Figure 5 [25,26], the nonlinear material properties are applied to PDMS (Neo-Hookean model) and copper (bilinear kinematic hardening model) and these models use more curves and straight lines connected to each other to describe more truthfully the material behavior. With different design routing patterns, the simulation result as shown in Figure 5b [25] tells the curve routing is much better than the others in the directional transition region.

The numerical modeling, as shown in Figure 5c [26], discusses the effect of the pitch on the mechanical behavior of the parallel aligned stretchable routing. The result in Figure 5d [26] shows that a smaller pitch will cause higher routing strain in parallel routing and the routing strain will be like that of single routing when the pitch is over 2.5 mm. This result tells us that the pitch of parallel routing must be considered in routing patterns.

**Figure 6.**
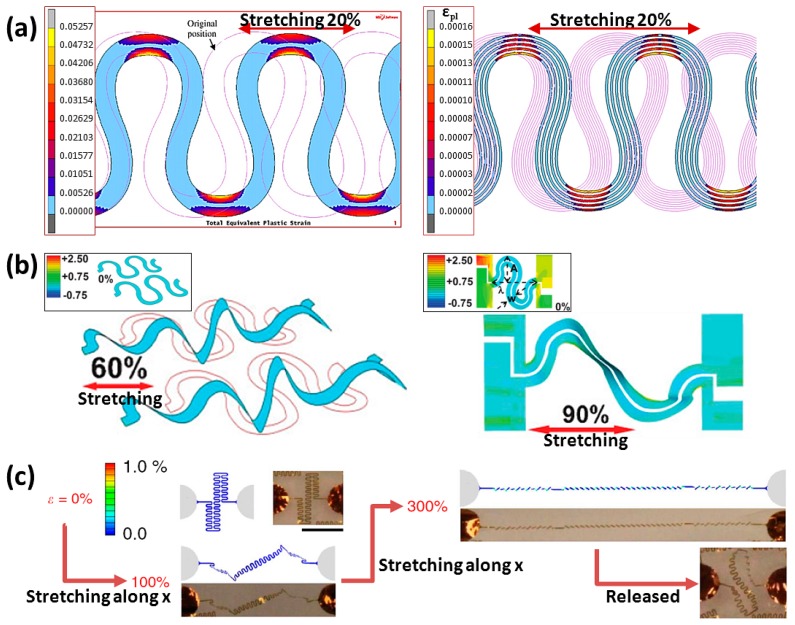
Simulation of stretchable routings in various situations. (**a**) Multi-tracked. Reproduced with permission from [27]; published by Elsevier, 2008. (**b**) Un-bonded. Reproduced with permission from [28]; published by John Wiley and Sons, 2009. (**c**) Without substrates (in the experimental conditions, the routing is embedded in a hollow elastomer filled with gel electrolyte). Reproduced with permission from [13]; published by Nature Publishing Group, 2013.

Multi-tracked routing design also helps a lot in decreasing routing strain as shown in Figure 6a [27]. The effect of decreasing the width of routing in a single-track in a previous nonlinear model as shown in Figure 4a [22] also finds the same trend. The stretchable routings embedded or bonded on substrates always limit the stretchability compared with the theoretical strain of a full-straightened shape. The simulation of routing in only bonding its electrodes is shown in Figure 6b [28], and it tells us that the mechanical behavior of a stretchable routing working in both coplanar and non-coplanar planes (island-bridge form, described in the next section) lead to excellent performance. The stretchable routing, embedded in a hollow elastomeric substrate which is filled with gel electrolyte, can show experimental behavior during tensile tests similar to the simulated result of routing stretching without substrates (Figure 6c) [13], and the routing pattern design finally reaches the theoretical strain although the routing pattern changes after releasing from 300% stretching.

The simulations above provide a clear view of how the parameters of routing design influence performance. Because of the physics limitations of simulation programs, it is difficult to build an approximate model of the conductor-composite wire system for simulation, and research on conductor-composite wires is always done by actual microfabrication. More routing design forms will be explained in the next section.

### 2.2. Fabrication Process

**Figure 7.**
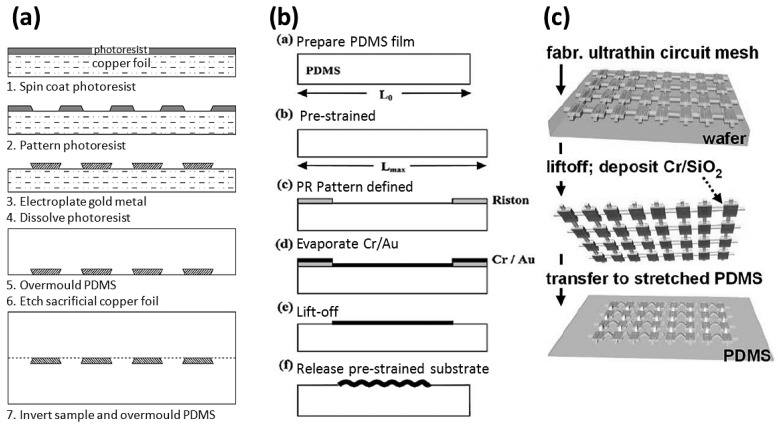
Three types of routing processes. (**a**) Coating silicon rubber on pre-prepared routings. Reproduced with permission from [19]; published by IEEE, 2007. (**b**) Patterning routings on pre-strained substrates. Reproduced with permission from [29]; published by IEEE, 2004. (**c**) Transferring pre-prepared routings on pre-strained substrates. Reproduced with permission from [30]; published by the National Academy of Sciences, USA, 2008.

To create stretchable electronics, fabrications can be roughly divided into three types as shown in Figure 7a–c [19,29,30]: coating low-modulus silicone rubber gel (e.g., PDMS) on pre-prepared wires, patterning metal materials as wires on prepared elastomeric substrates, and printing pre-prepared wires on also prepared stretchable substrates. Conductor-composed wires use many specific manufacture methods and we will describe them individually in a later section.

Before coating silicone rubber gel, the wires can be pre-produced on a carry wafer, and a sacrificial layer between them is then essential. The method is only used for wire design in a coplanar plane. Most simulations discuss the mechanical behavior and the almost maximal experimental performance has been found recently [18,19,22,25–27]. The advantage of the process is that it is easier to resist the residual stress of wires bonded onto stretchable substrates.

The process of patterning wires on initially prepared substrates can produce a non-coplanar routing because wires on pre-strained elastic substrates become wrinkly after releasing the substrate back to its natural state [29]. The process of depositing metal materials usually uses a shadow mask during e-beam evaporation. This method can produce a wavy-and-wrinkly routing bonded on elastomeric substrates.

As a routing-printing process, stretchable wires and elastomeric substrates are prepared separately. This could make a three-dimensional structured wire placed on a stretchable substrate if the wavy wires are transferred by printing onto pre-strained substrates [30]. Since it has the highest freedom for designing stretchable wires, the complexity is also markedly the highest among the three.

## 3. Forms of Stretchable Conductor

The classification method for elastic conductors, based on the composition of conductors (pure or compound) and the spatial relationship between routing and substrate (coplanar, non-coplanar, or mixed), can roughly divide stretchable routings into five forms: straight, wavy, wrinkly, island-bridge, and conductive-elastomeric. The material used in stretchable substrates is mostly polydimethylsiloxane (PDMS). PDMS is transparent, nonconductive, non-hydrophilic, and elastomeric. Its thermal endurance range is from −55 °C to 200 °C. After being bombarded by O_2_ plasma, PDMS becomes hydrophilic and can easily bond with silicon or macromolecule substrates by compression. Some bio-compatible materials can strongly bond with PDMS, e.g., glass, silicon, SiO_2_, Si_3_N_4_ and PDMS itself. Gold is usually chosen as the material for metal wires because of its high ductility, and copper is the second choice due to its low cost and good pre-fabricated foldable properties.

### 3.1. Straight Form

**Figure 8.**
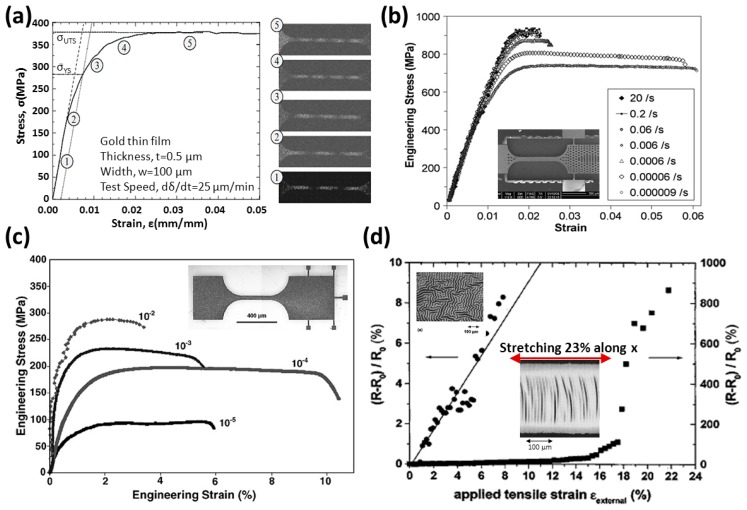
(**a**) Typical stress–strain curves and ESPI fringe patterns with various stress levels for Au thin film. Reproduced with permission from [31]; published by Springer, 2009. (**b**) The effect of varying tensile rate to stretch a gold wire. Reproduced with permission from [32]; published by Elsevier, 2010. (**c**) The effect of varying tensile rate to stretch a gold wire. Reproduced with permission from [24]; published by Elsevier, 2007. (**d**) The effect of different surface topography of gold wire in a tensile test. Reproduced with permission from [33]; published by AIP Publishing, 2003.

When beginning to study the relationship between the routing strain and the geometric routing design, one needs to examine the original tensile test for a micro-scale wire with straight form. According to the gold tensile test, as shown in Figure 8a, it is found that the failure strain of a gold wire with 0.5 μm thickness and 100 μm width is about 5% [31] and the strain-stress curve becomes flat after 2%. From the research results shown in Figure 8b, the gold wires with 0.85 mm thickness and 100 mm width will be broken with 2.5%–6% of routing strain which is dependent on the tensile rate [32]. The gold wire in Figure 8c has 2.8 μm thickness and 50 μm width and the failure strain is 4%–10.6% related with various tensile rates [24]. This tells us that a straight gold wire will break from 2% to 10% of tensile strain, which is high correlative with the tensile rate and the specimen thickness. If the tensile rate is much lower, most of gold straight wires could hold over 5% tensile strain, and break before 10%. The result is also proved in the left part of Figure 8d. The gold wire (0.1 μm thickness and 250 μm width) on PDMS substrates has ∼8% of failure strain, and the surface topography of routing is untidy due to the residual stress between gold and PDMS. This causes that the stress distribution of routing to not be uniform along the tensile direction, so some scientists have designed stripes into straight wires, like the stepladder shown on the right side of Figure 8d. After the residual stress is released, the shape of stripes becomes crescent-like. The striped wires can stretch up to 23%, albeit the rate of electrical resistance change begins to be unstable at 16% [33]. From those tests, it is said that the common allowable strain of a straight gold wire reaches 5%–10%, and it also could reach 16%–23% if the residual stresses between wires and substrates are released.

### 3.2. Wavy Form

**Figure 9.**
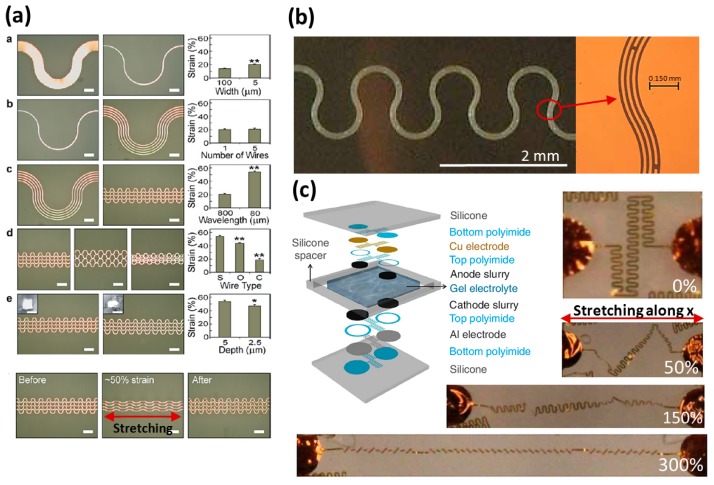
(**a**) Wavy elasticity conductors with various design parameters. Reproduced with permission from [18]; published by John Wiley and Sons, 2004. (**b**) Horseshoe-like wires by a multi-track design. Reproduced with permission from [19]; published by IEEE, 2007. (**c**) Mechanical behavior of a “self-similar” design of routing stretching from 0% to 300% of strain. Reproduced with permission from [13]; published by Nature Publishing Group, 2013.

According to the previous simulations described in Section 2, a wavy routing makes for more stretchability than a straight one. A research team produced a wavy routing (θ = 90°) by varying several essential routing parameters: width, wavelength, thickness, and pitch, to find the optimized performance [18]. The results are shown in Figure 9a and the phenomena deserve a detailed explanation.

#### 3.2.1. Width and Wavelength of Routing

The ratio of width and radius (half of wavelength), *W*/*R*, could be a basic design parameter: the smaller *W*/*R* of a routing set will cause a lower internal routing strain to occur during a tensile test. This increases the maximal allowable tensile strain of a wavy routing, as shown in part “a” of Figure 9a [18].

#### 3.2.2. Multi-Tracks of Routing

When reducing the *W*/*R* of routing (smaller width), it has more stretchability, but the routing also has higher electrical resistance and becomes easier to break. The design of multi-tracks can improve those two issues, and also keeps a similar stretchability [18].

#### 3.2.3. Thickness of Routing

As shown in part “e” of Figure 9a [18], a larger routing thickness provides more stretchability. When routings embedded in stretchable substrates are stretched, the substrates will displace the wires by vertical compressive stress. A wire with enough thickness can reduce the effect of the compressive stress and keeps its cross-section close to the original shape during stretching.

#### 3.2.4. Displacement from Substrates

The concept of compressive stress from substrates also explains why the wires with high *W*/*R* (1/40) cannot have higher allowable tensile strain compared to ones with lower *W*/*R* (1/4), although their thicknesses are the same, as shown in part “c” of Figure 9a [18]. The decreasing *W*/*R* of routing causes a lower routing deformation to occur during tensile tests. When the *W*/*R* of routing is too low as in a slender wavy wire, the pushing effect of compressive stress from substrates becomes stronger in both the coplanar and non-coplanar plane. The external stress from substrates will replace the internal stress of routing to destroy the wire. This tells us that there will be an optimized routing design *W*/*R* to get maximal performance. By the reference to Figure 9a, the optimized allowable routing design tensile strain is 54% ± 2% and almost reaches the theoretical strain of 57% [18].

#### 3.2.5. Angle of Routing

As shown in Figure 9b [19], another research team used a similar multi-track design and a wavy pattern to produce a horseshoe-like routing (θ = 120°) which increases the allowable tensile strain up to an average of 72%. Its theoretical strain according to Equation (1) is 142%, and the wire cannot stretch to reach the limit for the same reason known already—the pushing effect from stretchable substrates. This also tells that there will be an optimal routing design angle to get maximal performance, as shown in Figure 4c [18].

The stretchability improvement has one issue to be solved—the stress resulting from routings and substrates. A hollow elastomeric substrate was used to solve the problem. The stretchable substrate is combined with two silicone rubber films which have designed circuits transferred on them. The silicone spacer between two films supports the space for wire movement, and a gel electrolyte was injected to fill the space for maintaining the wires lightly in place, as shown in Figure 9c [13]. While stretching the device, the displacement effect from substrates will decrease to a much lower level, and the routing deformation can be considered as a situation of a wire stretching without substrates. From specific geometry design, the routing with up to 300% of allowable tensile strain is actually possible. The disadvantage of this device is gel electrolyte leakage.

### 3.3. Wrinkly Form

**Figure 10.**
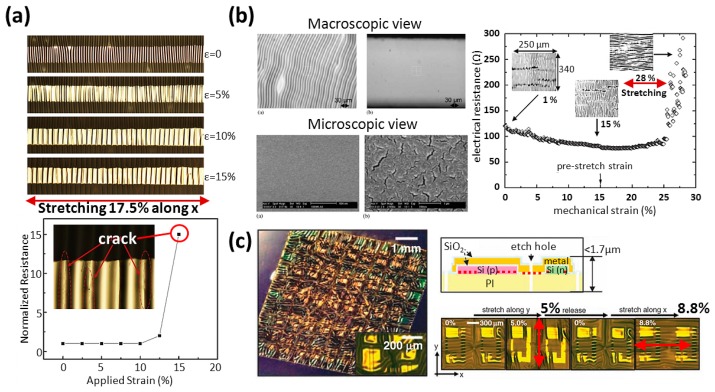
(**a**) Surface topography and normalized resistance of a wrinkly wire in a stretch test. Reproduced with permission from [21]; published by Elsevier, 2010. (**b**) SEM micrographs of wrinkly (left top) and flat (middle top) gold layers. The sample on the left is macroscopically buckled and that on the middle is flat. The buckled sample (left down) is continuous and has a grain-like structure, and the flat sample (middle down) surface presents a network of randomly arranged micro-cracks. Reproduced with permission from [20]; published by Elsevier, 2004. The different behaviors of pre-strained wire and straight wire in stretch test. Reproduced with permission from [29]; published by IEEE, 2004. (**c**) Stretchable silicon integrated circuits and the mechanical behaviors under 8.8% of tensile strain. Reproduced with permission from [34]; published by AAAS, 2008.

A wrinkly metal wire has good stretchability by extending its wavy structure in a non-coplanar plane. The fabrication commonly begins with preparing a pre-stretched substrate, followed by the patterning of wires using depositing-and-etching or evaporating through a shadow mask. Finally, releasing the pre-strained substrates and the residual stress of substrates makes the surface topography of routings in buckling. The process creates two advantages: residual stress release from substrates and increased theoretical strain of routings. The enhancing routing stretchability performance is dependent on the level of pre-strain of substrates. As shown in Figure 10a [21], the wire has 250 μm width and 5 nm/50 nm thickness of Cr/Au, and the substrates have a pre-stretched strain of 12.5%. A tensile test result tells us that the electrical resistance of routing stays stable before 12.5% where it increases wildly for the next 5%, and breaks finally at 17.5%. Considering the conclusions in Section 3.1 (straight form), the stretchability of this routing could easily be divided into two parts: 12.5% (pre-strained) and 5% (material property of gold wires). It is clear to observe how a wrinkly wire improves stretch-ability.

Releasing residual stress from substrates can improve the quality of wires. Because of the different thermal expansion coefficients of wires and substrates, the surface topography of routing has two types: wrinkled (compressive stress) or flat (tensile stress), and what type exists is dependent on the metal deposition conditions.

The wrinkled (or called buckled) and flat surface of wires are shown in Figure 10b [20,29]. Macroscopically, wrinkles distribute untidily along the pre-stretched axis, and Y-shaped wire cracks may occur during evaporation, even when mild heating is used during the processes. Microscopically, the buckled sample is continuous and has a grain-like structure, but the flat sample has randomly arranged micro-cracks. A wire with 500 mm width, a 5-nm thick Chromium (Cr) layer, a 25-nm thick gold (Au) layer, and a pre-strain of 15% can stretch up to 28%. The electrical resistance change of the wire during stretching also goes smoothly up to 15% and then it acts as a straight wire does. The maximal allowable strain of the routing finally reaches 28%.

**Figure 11.**
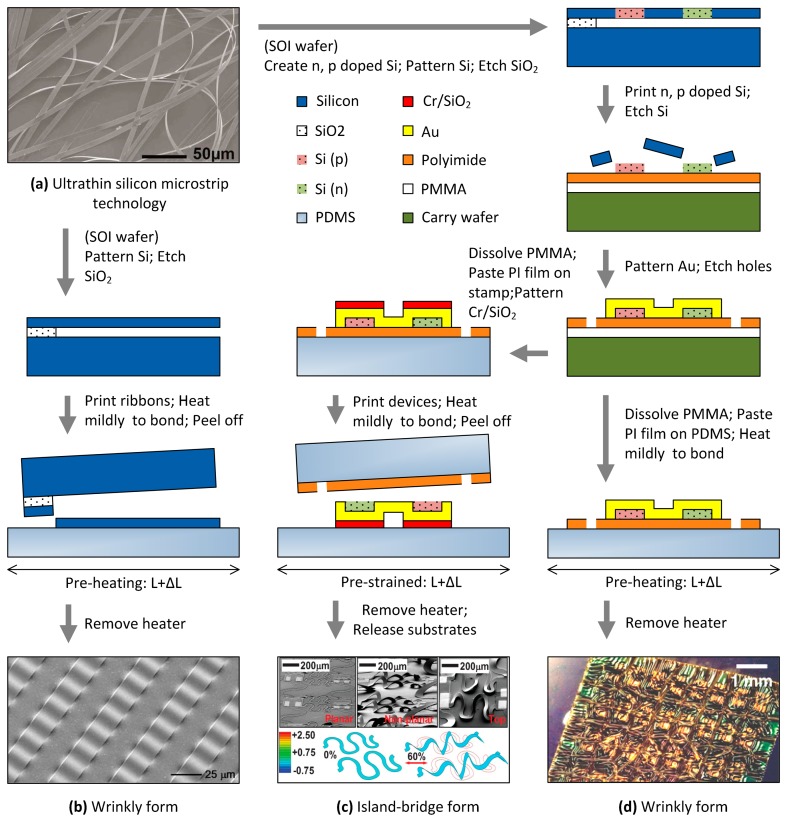
Process schematic of stretchable devices in various prototypes. (**a**) Ultrathin silicon microstrips. Reproduced with permission from [35]; published by AIP Publishing, 2004. (**b**) Silicon wrinkly strips. Reproduced with permission from [36]; published by AAAS, 2006. (**c**) Un-bonded stretchable integrated circuits. Reproduced with permission from [28]; published by John Wiley and Sons, 2009. (**d**) Wrinkly integrated circuits. Reproduced with permission from [34]; published by AAAS, 2008.

As shown in Figure 10c [34], a wrinkly polymer film (PI) with CMOS circuits is pasted on PDMS substrates. The routing pre-stretching process uses the mechanics of thermal expansion/contraction by placing the pre-heated PI on substrates and thereby makes circuits formed with a biaxial prestrain of around 5.7%. Since the fracture or plastic deformation in the PI is about 7%, the research team said the maximal failure strain of the device observed in experiments is around 10% plus the prestrain. Because the total thickness of polyimide (PI) including the integrated circuits (∼1.7 μm) and PDMS (∼700 μm) is very thin, the mechanical behavior of the devices through winds is almost like a soft flag. The same research group also reported progressively during 2004–2009 on a serious of microfabrication processes producing stretchable electronics [28,30,34–36], from the first step of making ultrathin silicon microstrips [35] to later stretchable integrated circuits [28,30], as shown in Figure 11a–d. Those processes make actual workable stretchable electronics in wrinkly or island-bride form and are worthy of a brief description.

Following the processes of Figure 11b [36], the researchers patterned silicon on Si-on-insulator (SOI) wafers, and etch the SiO_2_ layer to release ribbon-like thin silicon stripes. The silicon ribbons contact the pre-heating PDMS substrate (prestrain of 3%–5%) and both of them are heated continuously through a period of time. The SOI wafer is peeled off and will cause the ribbons to transfer onto PDMS substrates. Finally, the heater is removed and the substrates will shrink to make the ribbons buckle. The devices can be continuous with a strain of −10% to 5% in tensile and compression experiments. When they are doped with n/p type silicon and metal is patterned on the SOI wafer before patterning the silicon ribbons, the final product as a ribbon-like integrated circuit that can work under the tensile strain of −10%–10%. It is a basic design to make a real stretchable workable integrated circuit.

Since the process limits the geometric design of circuits, another process for printing circuits on PI substrates by following the steps of Figure 11d has been presented [34]. The silicon ribbons with n/p doped type silicon are pre-prepared, followed by printing them on the carrying wafer, which is coated with thin layers of polymethyl methacrylate (PMMA) (∼100 nm) and a PI layer (∼1.2 μm). After removing the unused silicon section by SF_6_ plasma etching, gold is patterned as wires and the empty region of PI is etched as many holes to help the dissolve the etchant into the sacrificial layer (PMMA). The PI substrate is released by dissolving the PMMA and pasted on the pre-heating PDMS substrates, followed by mild heating to bond. After removing the heater, the ultrathin PI film is finally buckled. The process following Figure 1c [28] will be presented in the next section because the device is made in island-bridge form.

The transfer technology of placing integrated circuits on stretchable substrates makes it possible to produce workable stretchable electronics. The methods have not only improved gradually in application through the years, but also solve the issue of connection between circuits and wires.

### 3.4. Island-Bridge Form

A wire moving comfortably without substrate limitations is defined as an island-bridge form, which could be combined with coplanar and non-coplanar routing designs. It can be produced by following the steps shown in Figure 11c [28,30]. The PI film with integrated circuits could be released by dissolving PMMA and then it is pasted on the stamp (PDMS). The metal layer (Cr/SiO_2_) for helping the circuits to adhere on PDMS substrates is patterned on a selected device area (except the wires) through a shadow mask. Then the integrated circuits are printed on pre-strained PDMS substrates by mildly-heating bonding. Because of the strong covalent bonding that forms between PDMS and SiO_2_, the integrated circuits can be transferred from PI film to the PDMS substrates. After removing the heater and pushing the device lightly, the wires will buckle like a bridge and peel off from PDMS substrates due to the lower adhesion force than other circuit parts (n,p type Si and electrodes).

**Figure 12.**
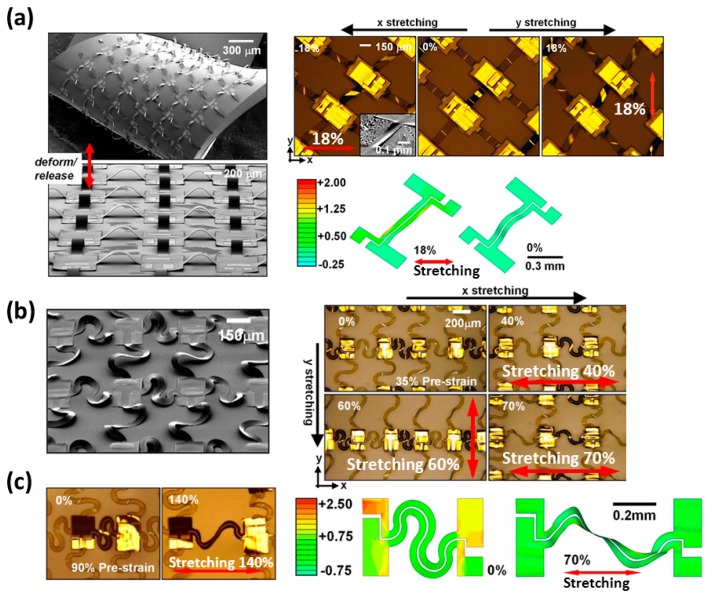
A stretchable array of CMOS inverters has (**a**) a prestrain of 17%; (**b**) a prestrain of 35%; and (**c**) a prestrain of 90%. Reproduced with permission from [30]; published by The National Academy of Sciences, U.S.A. 2008.

As shown in Figure 12a–c [30], the wires buckle as specific routing structures by the design of the researcher. The device with buckled straight routings in Figure 12a has a prestrain of 17% from substrates and can work under a tensile strain of 18%. The device with buckled wavy routings as shown in Figure 12b has a larger prestrain of 35% from substrates and can give an excellent stretchability of 70%. During a prestrain of 90% from substrates, the device in Figure 12c has an extremely high stretchable ability of 140%. The technology of making wires on both sides of simple supports can help stretchable electronics reach their design theoretical strain. After solving the issue of device protection in the working surrounding (for example: the hollow design in Figure 9c, the method could be reckoned as a final powerful tool for making stretchable integrated circuits.

### 3.5. Conductive-elastomeric Form

As the geometric routing designs on stretchable substrates have developed rapidly in the recent ten years, there is one more way to produce an elastic conductor. Scientists developed a specific form of routing an electrical conductor made by silicon rubber combined with amounts of electrical conductive particles [37–39]. Although there are many commercial products involving conductive rubber and pastes already sold on the market, the researchers studied on mixing process to keep improving the electrical conductivity and mechanical propriety of elastic conductors.

**Figure 13.**
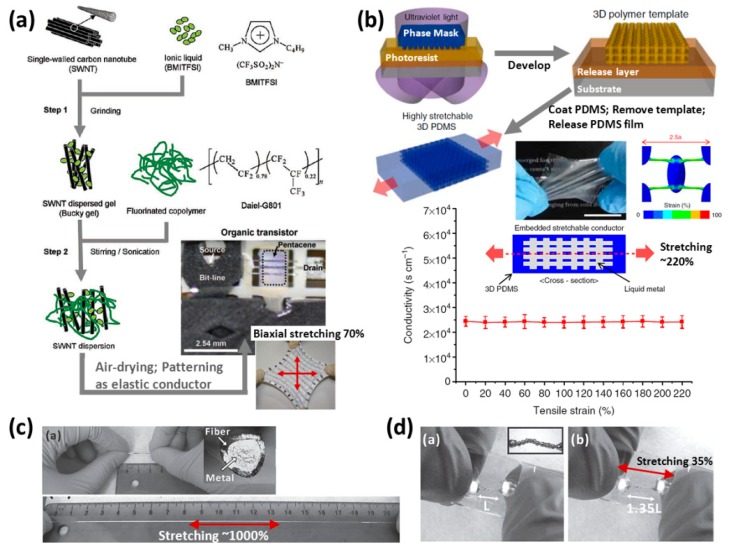
(**a**) Manufacturing process of SWNTs elastic conductor [37]. Reproduced with permission from [37]; published by AAAS, 2008. (**b**) An elastic conductor by using the technology of making three-dimensional nano-networks. Reproduced with permission from [40]; published by Nature Publishing Group, 2012. (**c**) An ultra-stretchable fiber with metallic conductivity. Reproduced with permission from [42]; published by John Wiley and Sons, 2012. (**d**) A stretchable wires made by 3D printing. Reproduced with permission from [43]; published by John Wiley and Sons, 2013.

Super-growth single-walled carbon nanotubes (SG-SWNTs) are used as a chemically stable and highly conductive dopant. As shown in Figure 13a [37], the ionic liquid 1-butyl-3-methylimidazolium bis(trifluoromethylsulfonyl)imide (BMITFSI) disperses SG-SWNTs uniformly by stirring at 25 °C for 16 h, and the generated mixture (SWNT dispersed gel) will be processed as a paste-like substance. A fluorinated copolymer (vinylidene fluoride-hexafluoropropylene) is added to that gel also by stirring at 25 °C for 16 h. The final mixture becomes a SWNT-rubber composite gel after air-drying for 6 h or an elastic conductor if the drying time is more than 12 h. During the experiments varying the weight ratio of SWNT content, it is found the elastic conductor with 20% SWNTs content doesn't show reduced mechanical flexibility and exhibits a conductivity of 57 siemens per centimeter (S·cm^−1^) and a stretchability of 134%. Considering commercially available carbon-particle-based conductive rubber, it has a conductivity of 0.1 S·cm^−1^ during a tensile test from 0 to 160% strain. This study improved the conductivity drastically.

Silver nanowires (AgNWs) are also gradually applied in making conductive elastomers. An AgNW/PDMS stretchable conductor with a stretchability of over 80% and a conductivity of 5285 S·cm^−1^ at 50% of tensile strain was created [38]. If two PDMS substrates with the same patterned conductive strips of AgNWs are combined with a middle layer of a biodegradable polymer (Ecoflex), it would form a wearable multifunctional monitoring device by capacitive sensing [39].

The manufacture of three-dimensional nanonetworks is applied to create a stretchable mesh-shaped wire embedded in elastomeric substrates, as shown in Figure 13b [40]. The technology of proximity-field nanopatterning (PnP) is the core of the study. PnP is a 3D nanofabrication technology involving a single-step exposure through a conformal phase mask, and it could produce hierarchical 3D photoresist (PR) nanostructures. The researchers used PnP to make a polymer template which has a 3D net-shaped periodic structure, and then infiltrate the PDMS prepolymer into the template, followed by an exposure again. After removing the polymer template and releasing layer in PR development, a PDMS substrate with 3D micro-channels inside is obtained.

The internal micro-channels of PDMS can support a deformation of over 250% of tensile strain. The liquid metal (eutectic gallium-indium, EGaIn, commonly consisting of 75% Ga 25% In by weight, 15.5 °C melt point [41]) has been injected into an elastomeric container made by enclosing two pieces of 3D micro-channels embedded in PDMS substrates as a final elastic conductor. The device has a stretchability of ∼220% and keeps the intrinsic conductivity of EGaIn along the whole tensile test history.

The liquid metal is also studied in an ultra-stretchable conductive fiber as shown in Figure 13c [42]. The scientists used a thermoplastic elastomer (poly[styrene-*b*-(ethylene-*co*-butylene)-*b*-styrene], SEBS) as a hollow fiber with a stretchability of 800%–1000%, followed by injecting liquid metal (EGaIn) into it. The conductive stretchable fiber can maintain an electrical continuity at ∼1000% of tensile strain with high electrical resistance, and this electrical resistance is a function of strain that grows linearly at 0%–600%. It's a novel design which has possibilities in wearable electronics applications, but the main issue is how to prevent the liquid metal from leaking.

3D printing is an increasingly popular technology. A study using EGaIn as the injecting material of 3D printing creates a stretchable conductive structure directly. As shown in Figure 13d [43], the injected wires covered by PDMS substrates can stretch up to 35% of tensile strain and maintain their electrical continuity. The research provides a simple way to make an arbitrary stretchable routing design. The conductive elastomer has an excellent stretchability compared with the stretchable routing in the other form. The integration between elastic conductors and precise circuits would be an issue worthy of study and development in the future.

## 4. Current and Future Developments

Stretchable electrical routing has become popular thanks to its widespread applications and strong support of flexible devices. Three aspects of its current and future development can be discussed.

### 4.1. Routing Design

Straight wires are less studied in this field, except for SMEA. The fabrication and design of wavy wires are reaching a state of maturity, and the peak strain during experiments is large enough to use in practice. The technology for fabrication of wrinkly wires is also stable enough for advanced design. The wires having two mixed geometric designs (serpentine and wrinkly) were just successfully produced and tested, but the concepts are listed in many patents. Wrinkle designs can support stable deformation when stretching or compressing, while wave designs can support higher stretchability. The more stretchability a wire has, and the less strain it has to bear and its conductivity will be more uniform in tensile tests. In another research concept, mixture designs got extra good results; but the process is not compatible with current CMOS fabrication technology. It may have a different potential in these fields.

For future development of routing design, the island-bridge design should be more studied. Although it has excellent ability now (0.5% wire strain needed when the substrate strain is up to 70% [30]), this performance is only possible without a sealer. For the condition of wires embedded in stretchable substrates, the gel sealing technology [13] could be more studied and tested.

### 4.2. Stretchable and Flexible Micro-Devices

Si-transistor and integrated circuits were produced and tested successfully. An existing issue can be the known unstable conductivity of wires and circuits when stretched. The problem must be considered carefully when they are applied in precise circuits, especially in sensing circuit designs. For sensing device design, one can consider them as flexible devices. Nowadays, many flexible devices are already made, such as tactile sensor arrays, photodetector arrays, thermal sensor array, flexible displays, but it is still a lack of research for building on stretchable substrates. In future developments, the integration of flexible functional devices, stretchable circuits, and elastomeric substrates may be recognized as an important topic.

### 4.3. Bonding, Assembling, and Packaging Technologies

Stretchable integrated circuits were successfully built on curvy objects [8] by similar technologies to those shown in Figure 11c. For assembling LEDs on stretchable substrates, some scientists have also studied and made prototypes [17]. In the future, a network node may have many functional devices, and some could be bought in market. How to assemble, bond, and pack those totally different devices (for use in energy harvesting, sensing, storing, processing, and communication) on one stretchable substrate is an existing challenge (a prototype has been described in [4]) and the technology will keep improving in the long-term in future developments of stretchable electrical devices.

## 5. Conclusions

In this review, the authors present the typical wire designs, simulations and the associated concepts in this field. For lots of studies published around the world today, the development of stretchable routings is just about to take off into a larger domain, such as device integration, reliability test, and stable performance in precise circuits. In the future, one can imagine many broad applications: pasting an ultrathin electrical skin on our body for daily health monitoring; high-resolution electronic eyeballs the help the blind see the beautiful planet; a hand prosthesis built with tactile and thermal sensing functions that works like a real hand; after watching newspapers on portable electronic devices, people could fold the device and put it in their pockets; or especially, a personal flexible notebook that people can just roll up and take away.
